# ﻿New species and illustrated key of *Macraspis* (Scarabaeidae, Rutelinae, Rutelini) from the Amazon biome of Brazil

**DOI:** 10.3897/zookeys.1124.91156

**Published:** 2022-10-17

**Authors:** Matheus Bento, Mary Liz Jameson, Matthias Seidel

**Affiliations:** 1 Instituto Nacional de Pesquisas da Amazônia (INPA), Coordenação de Biodiversidade, Laboratório de Sistemática e Ecologia de Coleoptera (LASEC), Manaus, Brazil Instituto Nacional de Pesquisas da Amazônia Manaus Brazil; 2 Wichita State University, Wichita, Kansas, USA Wichita State University Wichita United States of America; 3 Naturhistorisches Museum Wien, Zweite Zoologische Abteilung, Burgring 7, 1010 Vienna, Austria Naturhistorisches Museum Wien Vienna Austria

**Keywords:** Chafers, identification, morphology, Neotropical Region, taxonomy

## Abstract

The phytophagous scarab genus *Macraspis* MacLeay (Scarabaeidae, Rutelinae, Rutelini) is reviewed from the Brazilian Amazon region. Three new species are described and illustrated from the states of Amazonas, Pará, and Rondônia: *M.buehrnheimi***sp. nov.**, *M.opala***sp. nov.**, and *M.phallocardia***sp. nov.** Two species, *Macraspisfernandezi* Neita-Moreno and *M.oblonga* Burmeister, are recorded for the first time in Brazil (new country records). *Macraspismaculatacrosarai* Soula is a new synonym of *Macraspismaculata* Burmeister; hence this species no longer includes subspecies. Furthermore, *Macraspiscinctaparensis* Soula, 2005 is deemed unavailable under the provisions of ICZN Articles 16.4.1 and 16.4.2. An illustrated key to 15 species and subspecies of *Macraspis* from the Brazilian Amazon enables identification of this speciose leaf chafer genus.

## ﻿Introduction

**Figure 1.**
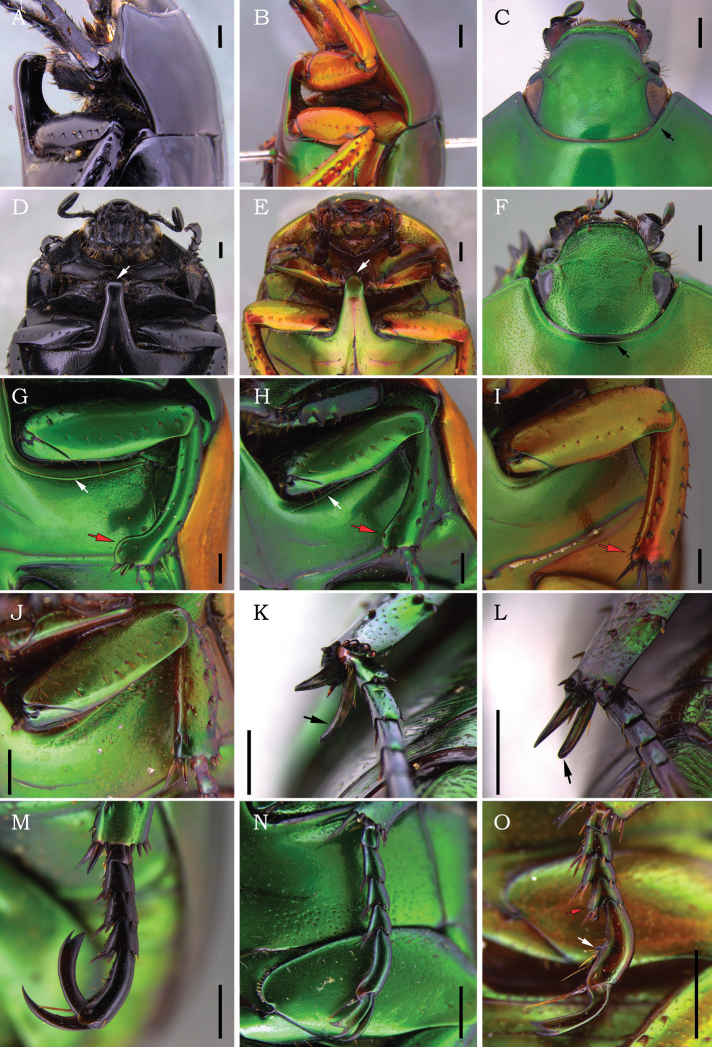
*Macraspis* spp. (**A–O**). Lateral and ventral views of pro- and mesothorax of **A, B***M.morio* and **D, E***M.p.pseudochrysis*. Dorsal view of head and anterior portion of pronotum of **C***M.festiva* and **F***M.c.chloraspis*. Anterior view of mesothoracic legs of **G***M.festiva***H***M.oblonga***I***M.p.pseudochrysis* and **J***M.c.chloraspis*. Female metatibial spurs of **K***M.c.chloraspis* and **L***M.phallocardia* sp. nov. Anterior view of mesotarsus of **M***M.p.pseudochrysis***N***M.c.chloraspis* and **O***M.fernandezi*. Scale bars: 1 mm.

**Figure 2.**
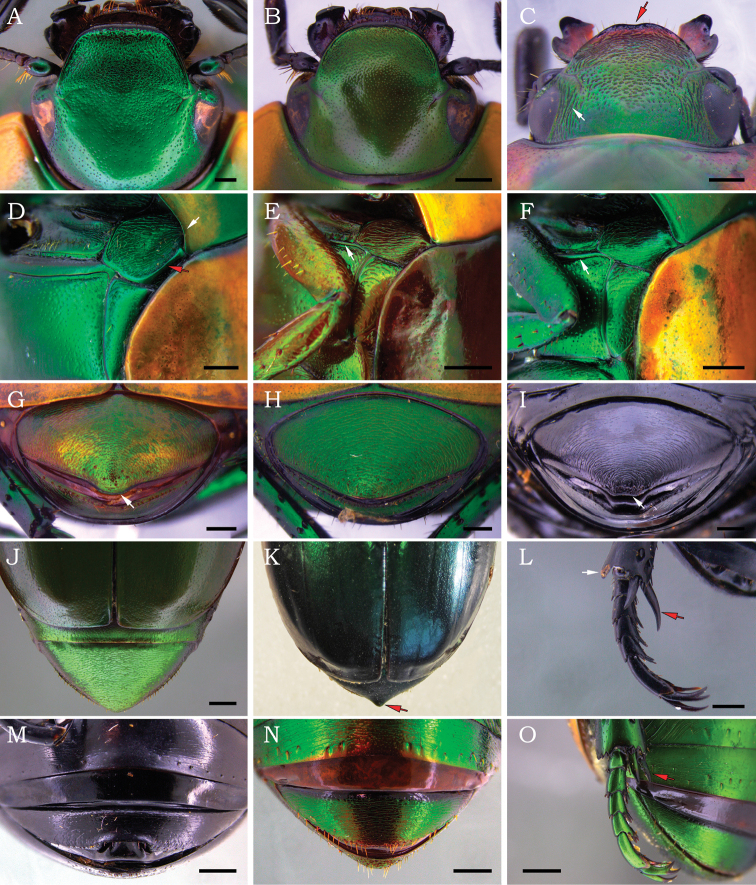
*Macraspis* spp. (**A–O**). Dorsal view of head of **A***M.oblonga***B***M.olivieri* and **C***M.xanthosticta*. Lateral view of mesothorax of **D***M.festiva***E***M.maculata* and **F***M.c.chloraspis*. Female pygidium of **G***M.oblonga***H***M.olivieri* and **I***M.morio*. Dorsal view of female abdomen of **J***M.p.pseudochrysis* and **K***M.lepiouffi* paratype. Metatarsus of **L***M.morio* and **O***M.olivieri*. Female last abdominal ventrites of **M***M.morio* and **N***M.p.pseudochrysis*. Scale bars: 0.5 mm (**A–C**), 1 mm (**D–O**).

**Figure 3.**
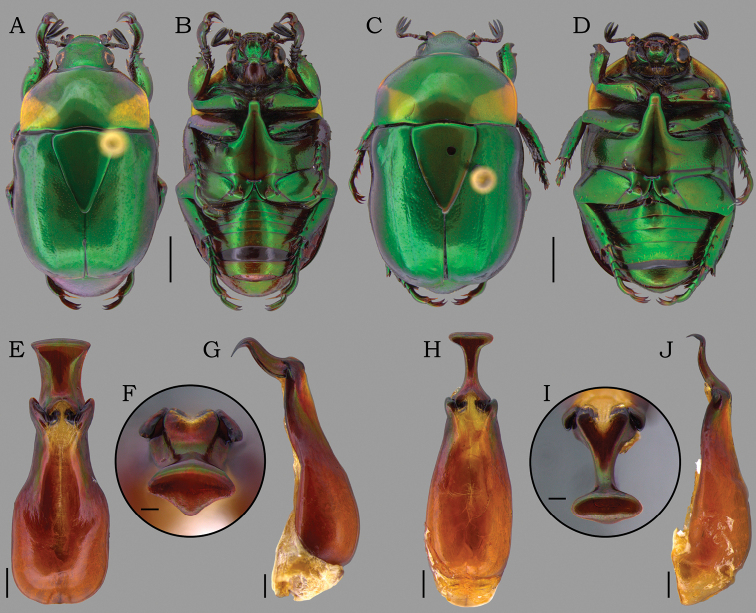
*Macraspisbuehrnheimi* sp. nov. (**A–G**) and *Macraspisfernandezi* Neita-Moreno, 2014 (**H–J**) *Macraspisbuehrnheimi* sp. nov. **A** dorsal view of holotype male and **B** ventral view of holotype male **C** dorsal view paratype female and **D** ventral view view paratype female **E–F** aedeagus of holotype (**E** dorsal **F** caudal **G** lateral views). *Macraspisfernandezi* Neita-Moreno, 2014 from Caracaraí, Roraima state, Brazil (new country record) **H** aedeagus dorsal view **I** aedeagus caudal view and **J** aedeagus lateral view. Scale bars: 2 mm (**A–D**); 0.5 mm (**E, G, H, J**); 0.2 mm (**F, I**).

**Figure 4.**
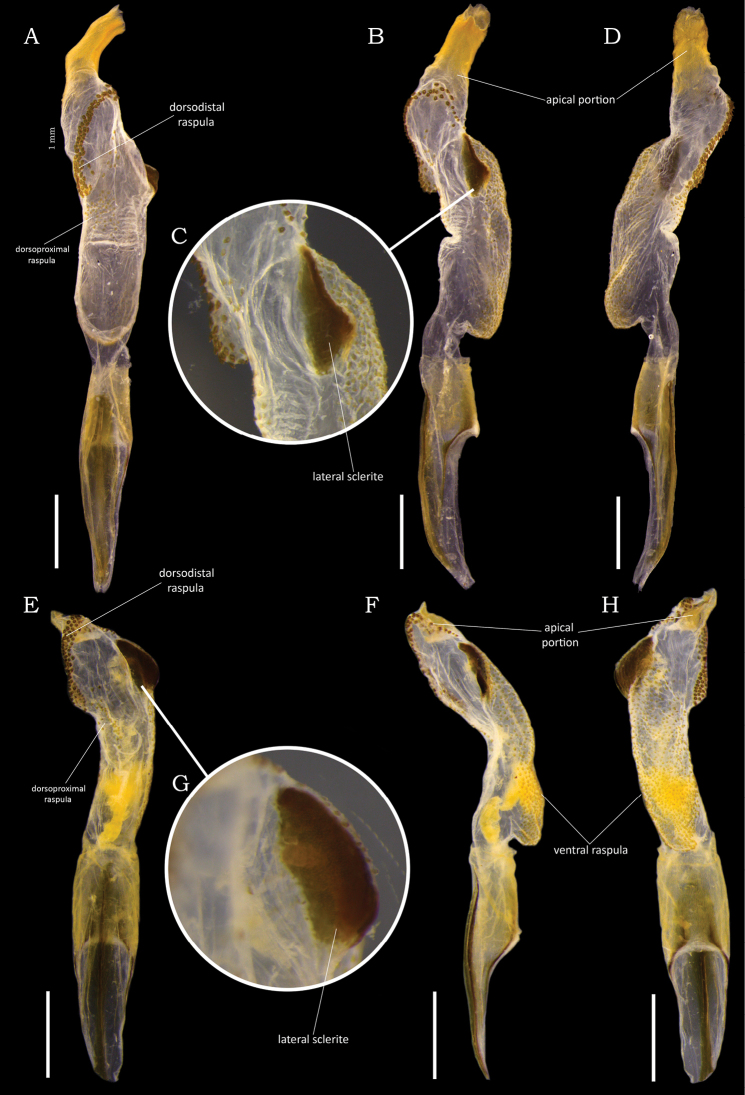
Comparison of male endophalli of *Macraspisbuehrnheimi* sp. nov. and *Macraspisfernandezi* Neita-Moreno, 2014. *Macraspisbuehrnheimi* sp. nov. (**A–D**) in **A** dorsal **B** lateral and **D** lateral views, with **C** detail showing lateral sclerite. *Macraspisfernandezi* Neita-Moreno, 2014 (**E–H**) in **E** dorsal **F** lateral and **H** ventral views, with **G** detail showing lateral sclerite. Scale bars: 0.5 mm.

**Figure 5.**
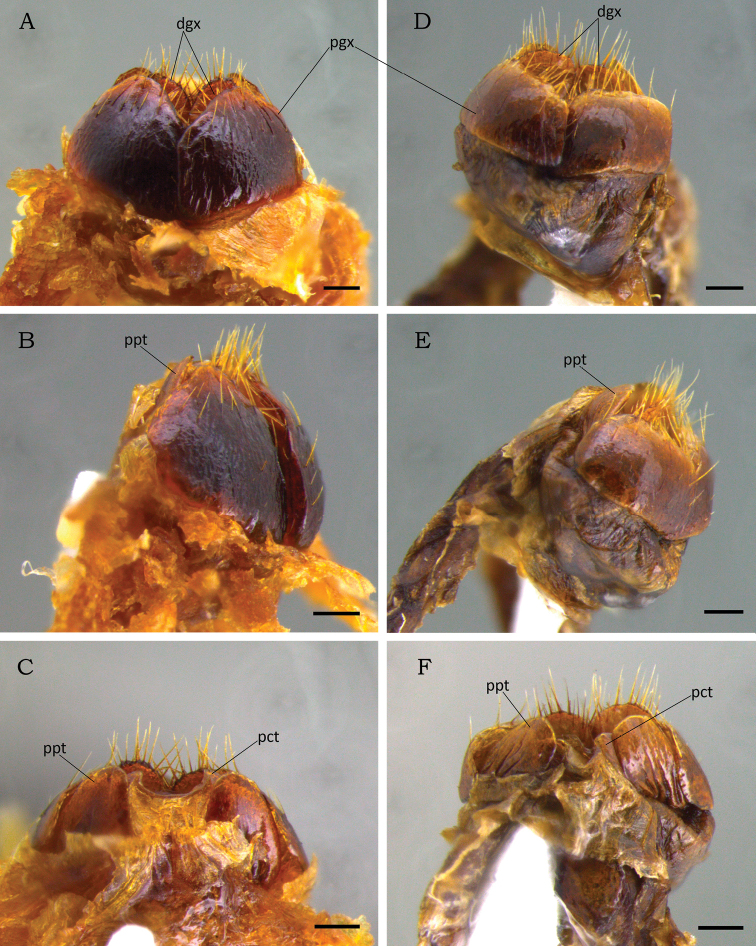
Comparison of female external genitalia of *Macraspisbuehrnheimi* sp. nov. (**A–C**) and *Macraspisphallocardia* sp. nov (**D–G**). *Macraspisbuehrnheimi* sp. nov. in **A** ventral **B** lateral and **C** dorsal views. *Macraspisphallocardia* sp. nov. in **D** ventral **E** lateral and **F** dorsal views. Abbreviations: dgx = distal gonocoxite; pgx = proximal gonocoxite; pct = proctiger; ppt = paraproct. Scale bars: 0.3 mm.

The Neotropical genus *Macraspis* MacLeay, 1819 (Scarabaeidae, Rutelinae, Rutelini) is a distinctive and widely distributed leaf chafer group that occurs from Mexico to Argentina (Soula 1998). This genus is diagnosed by (1) mandibles with outer margin bidentate (Fig. 2C), (2) pronotum evenly trisinuated posteriorly and with a concave posteromedial emargination (Fig. 3C), (3) scutellar shield as long as the elytral suture (Fig. 3C), (4) mesometaventral process well developed (Fig. 1A, D), and (5) femur-abdominal stridulatory apparatus present (Fig. 1K) (Ohaus 1903; Soula 1998).

We examined the *Macraspis* species and subspecies with distributions in the Brazilian Amazon biome (defined as northern Brazil including the states of Acre, Amapá, Amazonas, Pará, Rondônia, Roraima, as well as small portions of Mato Grosso, Tocantins, and Maranhão states) (IBGE 2004) (Fig. 12) and included them in the identification key. Species from the Atlantic Forest, Caatinga, Pampa, Pantanal, or Cerrado biomes in Brazil are not part of our research focus.

*Macraspis* currently includes 68 species and 21 subspecies (Soula 1998, 2002, 2005, 2006, 2010; Moore et al. 2014; Neita-Moreno 2014; Bento and Grossi 2021). As a result of our work, 15 species and subspecies are known from the Brazilian Amazon. The study of the taxa in Brazil led to the discovery of three undescribed species of *Macraspis* from two different Amazonian interfluvial areas, as well as two species that are new country records in Brazil: *M.fernandezi* Neita-Moreno, 2014 and *M.oblonga* Burmeister, 1844. Based on comparative examinations of type and non-type specimens, we synonymize *M.maculatacrosarai* Soula, 1998 with *Macraspismaculata* Burmeister, 1844. Under the provisions of ICZN (1999) Articles 16.4.1 and 16.4.2, *M.cinctaparensis* Soula, 2005 is an unavailable name. As a result of these changes, the genus *Macraspis* now includes 71 species and 19 subspecies. We present an illustrated key to all species and subspecies from Brazilian Amazon biome, and this key provides a foundation for broader identification of this speciose leaf chafer genus.

## ﻿Methods

The type material and additional specimens used for descriptions, comparisons, and key are deposited in the following institutions (acronym and curators parenthesized): Seção de Entomologia da Coleção Zoológica da Universidade Federal do Mato Grosso, Cuiabá, Brazil (**CEMT**; Fernando Zagury Vaz-de-Mello); Coleção Entomológica do Instituto Oswaldo Cruz, Rio de Janeiro, Brazil (**CEIOC**; Márcio Felix, Claudia Leal Rodrigues); Musée des Confluences, Lyon, France (**CCECL**; Cédric Audibert); Coleção Zoológica Prof. Paulo Bührnheim, Universidade Federal do Amazonas, Manaus, Brazil (**CZPB**; Fábio Siqueira Pitaluga de Godoi); Coleção Entomológica Pe. Jesus Santiago Moure, Universidade Federal do Paraná, Curitiba, Brazil (**DZUP**; Lúcia Massutti de Almeida); Colección Entomológica del Instituto Alexander von Humboldt, Bogotá, Colombia (**IAVH**; Jhon César Neita-Moreno); Coleção de Invertebrados do Instituto Nacional de Pesquisas da Amazônia, Manaus, Brazil (**INPA**; Márcio de Oliveira); Naturhistorisches Museum Wien, Vienna, Austria (**NHMW**; Matthias Seidel); Mary Liz Jameson Collection, Wichita, Kansas, USA (**MLJC**), Martin-Luther-Universität, Zentralmagazin Naturwissenschaftlicher Sammlungen, Zoologische Sammlung, Halle, Germany (**MLUH**; Hendrik Müller); Muséum national d’Histoire Naturelle, Paris, France (**MNHN**; Antoine Mantilleri); Coleção Entomológica ‘Prof. J.M.F. Camargo’, Departamento de Biologia, Faculdade de Filosofia, Ciências e Letras – USP, Ribeirão Preto, Brazil (**RPSP**; Eduardo Andrade Botelho de Almeida); Zoologisches Museum, Universität Kiel, Kiel, Germany (**ZMUK**; Michael Kuhlmann); Wichita State University Invertebrate Collection, Wichita, KS, USA (**WICHI**).

Body length was measured from the apex of the clypeus to the apex of the elytra; body width was measured across the humeri. Regarding punctures, surfaces were considered punctostriate when punctures were confluent and elongated, densely punctate if punctures were nearly confluent to less than two puncture diameters apart, moderately punctate if 2–6 punctures diameters apart, and sparsely punctate if punctures were separated by more than six puncture diameters. The punctures were considered small when less than 0.019 mm in diameter, moderate when 0.02–0.049 mm, and large if larger than 0.05 mm. Setae were defined short if between 0.1–0.19 mm in length, moderately long if between 0.2–0.39 mm, and long if longer than 0.4 mm. Regarding density of setae, surfaces were considered densely setose when many setae completely covered the surface, moderately setose when the surface visible and with many setae, and sparsely setose when the surface was visible and with only a few setae.

Morphological terms follow Beutel and Lawrence (2005) for the general morphology and the male and female genitalia (with the adoption of the term tectum for the distal portion of the phallobase [non-apodeme]), and d’Hotman and Scholtz (1990) and Coca-Abia and Martín-Piera (1991) for endophallus structures.

Like many other *Macraspis* species, identification of the two new species described here requires examination of male genitalia. Female external genitalia of these new species showed conspicuous differences in the proximal and distal gonocoxites. However, caution must be taken concerning the identification of females based only on external genital characters because the whole range of intraspecific variation is not known for these structures. Accordingly, females of these species can be more reliably identified when collected with associated males. Therefore, females were not included in the diagnoses and key.

For purposes of the identification key, we include species known to occur only within the Brazilian Amazon biome (Soula 1998, 2002). The key as presented here does not include females of *M.lateralis* (Olivier, 1789) because of scarce specimens as well as the lack of associated males. Further advances must await additional female specimens and more morphological studies including type material to diagnose the female of *M.lateralis* and identify the hitherto unknown female of *M.fernandezi*.

Images were taken using a Leica DFC295 camera attached to a Leica M165C stereomicroscope and were processed using the Leica Application Suite (LAS) v. 4.1 and Helicon Focus (HeliconSoft) software. The photographic illumination follows Kawada and Buffington (2016).

The verbatim label data from type specimens are transcribed in quotation marks, with “/” used to separate lines on the same label, and “//” to separate labels. Label data from non-type specimens are provided as follows: country, state or province, locality, date, collector or old collection (quantity, sex symbol, collection acronym).

The geographic coordinates were obtained with Google Maps and the georeferenced points were plotted on the distribution map generated by the web software Simplemappr (Shorthouse 2010).

## ﻿Results

Based on examination of specimens in 12 collections, we recorded seven species as occurring in the Brazilian Amazon: *M.buehrnheimi* sp. nov., *M.fernandezi* Neita-Moreno, *M.lateralis* (Olivier), *M.martineziauzerali* Soula, 1998, *M.oblonga* Burmeister, *M.opala* sp. nov., and *M.phallocardia* sp. nov. We provide species treatments and a distribution map for all species including *M.maculata* Burmeister, 1844 (Fig. 12). Eight additional species are known to occur in the Amazon biome: *M.chloraspischloraspis* Laporte, 1840, *M.festiva* Burmeister, 1844, *M.lepiouffi* Soula, 1998, *M.morio* Burmeister, 1844, *M.olivieri* (Waterhouse, 1881), *M.peruviana* Ohaus, 1898, *M.pseudochrysispseudochrysis* Landin, 1965, *M.xanthosticta* Burmeister, 1844. As a result of our research, *M.maculatacrosarai* Soula is synonymized with *M.maculata* Burmeister. The taxon *sensu*Soula (1998, 2003) is considered endemic to the Brazilian Cerrado and Atlantic Forest rather than the Brazilian Amazon. For this reason, this species was excluded from the key. All species and subspecies recorded from the Brazilian Amazon region are included in the identification key to allow for identification and comparison with other *Macraspis*.

*Macraspiscinctaparensis* Soula, 2005 was described based on two specimens with the doubtful locality of Obidos, Pará state, Brazil. In the original description Soula (2005) stated: “I dare to hope that it is not a labeling error on the locality” (translated from French). Indeed, Obidos is a historically highly frequented collecting site, and *Macraspiscincta* was never reported from that location. Additionally, all *Macraspiscincta* subspecies occur exclusively in the Atlantic Forest from the Brazilian states of Rio de Janeiro, São Paulo, Espírito Santo, and Santa Catarina. Furthermore, the original description violates the ICZN (1999) Articles 16.4.1 and 16.4.2 stating that every new specific and subspecific name published after 1999 must be accompanied in the original publication “by the explicit fixation of a holotype, or syntypes, for the nominal taxon” and “where the holotype or syntypes are extant specimens, by a statement of intent that they will be (or are) deposited in a collection and a statement indicating the name and location of that collection”. Because Soula (2005) did not provide a type designation and collection information, *M.c.parensis* is unavailable.

### ﻿Key to Brazilian Amazon species and subspecies of *Macraspis*

Lacking females of *M.buehrnheimi* sp. nov., *M.fernandezi*, *M.lateralis*, *M.opala* sp. nov., *M.phallocardia* sp. nov.

**Table:** 

1	Protarsomere V enlarged; anterior protarsal claw thickened and unequally cleft; pygidium strongly convex, with posterior margin ventrally positioned, male	**2**
–	Protarsomere V not enlarged; anterior protarsal claw not thickened and equally cleft; pygidium weakly convex to plano-convex, with posterior margin dorsoapically positioned, female	**16**
2	Mesometaventral process thickened and oblique in relation to the longitudinal axis of the body (Fig. 1A), with apex expanded and bluntly rounded in ventral view (Fig. 1D); apical margin of metatibia strongly projected externally (Fig. 2L); inner metatibial spur distinctly longer than metatarsomeres I and II combined (Fig. 2L)	***M.morio* Burmeister**
–	Mesometaventral process weakly flattened and parallel in relation to the longitudinal axis of the body (Fig. 1B), with apex abruptly narrowed in ventral view (Fig. 1E); apical margin of metatibia weakly projected externally (Fig. 2O); inner metatibial spur subequal or shorter than metatarsomeres I and II combined (Fig. 2O)	**3**
3	Large body size (length 14–23 mm); head without supraorbital strigae (Fig. 2A, B); pronotum strongly emarginated posterolaterally (Fig. 2D); anterior bead of pronotum medially effaced or barely defined (Fig. 1C); mesotibia somewhat arcuate and as long as mesofemur, with an internoapical lobe weakly to strongly developed (Fig. 1G–I); mesepimeron ventrally concave and with a posterolateral carina (Fig. 2D)	**4**
–	Small body size (length 7–14 mm); head with supraorbital strigae (Fig. 2C); pronotum not emarginate or weakly emarginated posterolaterally (Fig. 2E, F); anterior bead of pronotum complete and well defined (Fig. 1F); mesotibia straight and distinctly shorter than mesofemur, with no internoapical lobe (Fig. 1J); mesepimeron slightly convex ventrally and without carina (Fig. 2E, F)	**9**
4	Mesotibia medially wider than half the mesofemur width (Fig. 1I); anterior meso- and metatarsal claw thick and narrowly cleft, and in flexed position distinctly longer than tarsomere V (Fig. 1M)	**5**
–	Mesotibia medially narrower than half the mesofemur width (Fig. 1G, H); anterior meso- and metatarsal claw flat and broadly cleft, and in flexed position subequal or shorter than tarsomere V (Fig. 1N)	**6**
5	Body non-metallic green; posterior border of mesocoxal cavity narrow (Fig. 1I); mesotibia with internoapical lobe inconspicuous (Fig. 1I); mesepimeron barely concave ventrally	***M.pseudochrysispseudochrysis* Landin**
–	Body metallic green; posterior border of mesocoxal cavity wide (Fig. 1G); mesotibia with internoapical lobe strongly developed (Fig. 1G); mesepimeron distinctly concave ventrally	***M.lepiouffi* Soula**
6	Dorsum unicolored, with elytra uniformly green and without metallic maculation	***M.peruviana* Ohaus**
–	Dorsum bicolored, with elytra yellow with or without metallic green maculation	**7**
7	Pronotum entirely green (lateral margins same color as disc)	***M.festiva* Burmeister**
–	Pronotum with disc green and lateral margins yellow	**8**
8	Clypeus rugopunctate at apex, with anterior margin strongly raised and subtruncated (Fig. 2A); scutellar shield shorter than elytral suture; posterior border of mesocoxal cavity narrow, with postcoxal line somewhat straight (Fig. 1H); internoapical lobe of mesotibia weakly developed and barely defined (Fig. 1H)	***M.oblonga* Burmeister**
–	Clypeus densely punctate at apex, with anterior margin weakly raised and rounded (Fig. 2B); scutellar shield longer than elytral suture; posterior border of mesocoxal cavity wide, with postcoxal line curved (Fig. 1G); internoapical lobe of mesotibia strongly developed and well defined (Fig. 1G)	***M.olivieri* (Waterhouse)**
9	Mesotibia medially narrower than mesofemur at middle (Fig. 1J); mesotarsomere IV with ventroapical process pointed and straight (Fig. 1N); mesotarsomere V ventrally untoothed and distinctly shorter than mesotarsomeres I–IV combined (Fig. 1N); anterior meso- and metatarsal claw broadly cleft and in flexed position shorter than metatarsomere V (Fig. 1N)	***M.chloraspischloraspis* Laporte**
–	Mesotibia medially as wide as mesofemur at middle (Fig. 2E); mesotarsomere IV with ventroapical process thick and ventrally swollen (Fig. 1O); mesotarsomere V with a ventromedial tooth and as long as mesotarsomeres I–IV combined (Fig. 1O); anterior meso- and metatarsal claw narrowly cleft and in flexed position longer than metatarsomere V (Fig. 1O)	**10**
10	Paramera apically dilated (Fig. 1E–J)	**11**
–	Paramera apically narrowed (Figs 3E–L, 4E–J)	**12**
11	Lateral articular areas of tectum truncated and weakly projected distally (Fig. 3H–J); paramera in dorsal view with middle portion strongly constricted and narrower than half the apical portion (Fig. 3H)	***M.fernandezi* Neita-Moreno**
–	Lateral articular areas of tectum pointed and strongly projected distally (Fig. 3E–G); paramera in dorsal view with middle portion slightly constricted and distinctly wider than half the apical portion (Fig. 3E)	***M.buehrnheimi* Bento, Jameson & Seidel, sp. nov.**
12	Apex of pygidium smooth (Fig. 9C); paramera laterally excavated (Fig. 9G)	***M.opala* Bento, Jameson & Seidel, sp. nov.**
–	Apex of pygidium with strong, concentric sculpturing (Fig. 9M); paramera laterally not excavated (Fig. 10G, J)	**13**
13	Smaller specimens (length 7–9 mm); anterior margin of clypeus weakly notched medially (more evident in females) (Fig. 2C); scutellar shield slightly constricted basolaterally; paramera with apex acute	***M.xanthosticta* Burmeister**
–	Larger specimens (length 10–11.7 mm); anterior margin of clypeus not notched; scutellar shield strongly constricted basolaterally; paramera with apex narrowly rounded or parabolic	**14**
14	Elytron metallic green with two median, yellow maculae or a single transverse yellow band	***M.martineziauzerali* Soula**
–	Elytron usually metallic green and without yellow maculae or band	**15**
15	Lateral articular areas of tectum thickened and deflected outward (Fig. 10E); paramera in caudal view rounded-oval, with sides not declivous and rounded to slightly constricted apically (Fig. 10F)	***M.phallocardia* Bento, Jameson, Seidel, sp. nov**
–	Lateral articular areas of tectum compressed and straight (Fig. 10H); paramera in caudal view laterobasally projected backward, with sides slightly declivous and narrowly constricted apically (Fig. 10I)	***M.lateralis* (Olivier)**
16	Mesometaventral process thickened and oblique to the body’s longitudinal axis (Fig. 1A), with apex expanded and bluntly rounded in ventral view (Fig. 1D); apical margin of metatibia strongly projected externally (Fig. 2L); inner metatibial spur distinctly longer than metatarsomeres I and II combined (Fig. 2L); posterior margin of pygidium medially truncated or slightly bidentate (Fig. 2I); ventrite 6 posteromedially emarginated (Fig. 2M)	***M.morio* Burmeister**
–	Mesometaventral process weakly flattened and parallel to body longitudinal axis (Fig. 1B), with apex abruptly narrowed in ventral view (Fig. 1E); apical margin of metatibia weakly projected externally (Fig. 2O); inner metatibial spur subequal or shorter than metatarsomeres I and II combined (Fig. 2O); posterior margin of pygidium medially rounded (Fig. 2G, H); ventrite 6 not emarginated (Fig. 2N)	**17**
17	Large specimens (length 14–23 mm); head without supraorbital strigae (Fig. 2A, B); pronotum strongly emarginated posterolaterally (Fig. 2D); anterior bead of pronotum medially effaced or barely defined (Fig. 1C); mesotibia somewhat arcuate and as long as mesofemur, with an internoapical lobe inconspicuous to strongly developed (Fig. 1G–I); mesepimeron ventrally concave and with a posterolateral carina (Fig. 2D)	**18**
–	Small specimens (length 7–14 mm); head with supraorbital strigae (Fig. 2C); pronotum weakly or not emarginated posterolaterally (Fig. 2E, F); anterior bead of pronotum complete and well defined (Fig. 1F); mesotibia straight and distinctly shorter than mesofemur, with no internoapical lobe (Fig. 1J); mesepimeron slightly convex ventrally and without carina (Fig. 2E, F)	**23**
18	Mesotibia medially wider than half the mesofemur width (Fig. 1I)	**19**
–	Mesotibia medially narrower than half the mesofemur width (Fig. 1G, H)	**20**
19	Body non-metallic green; pygidium apically obtuse in dorsal view (Fig. 2J); posterior border of mesocoxal cavity narrow (Fig. 1I); mesotibia with internoapical lobe inconspicuous (Fig. 1I); mesepimeron barely concave ventrally	***M.pseudochrysispseudochrysis* Landin**
–	Body metallic green; pygidium apically acuminate in dorsal view (Fig. 2K); posterior border of mesocoxal cavity wide (Fig. 1G); mesotibia with internoapical lobe strongly developed (Fig. 1G); mesepimeron distinctly concave ventrally	***M.lepiouffi* Soula**
20	Dorsum unicolored, with elytra uniformly green and without metallic maculation	***M.peruviana* Ohaus**
–	Dorsum bicolored, with elytra yellow with or without metallic green maculation	**21**
21	Pronotum entirely green (lateral margins same color as disc)	***M.festiva* Burmeister**
–	Pronotum with disc green and lateral margins yellow	**22**
22	Clypeus rugopunctate at apex, with anterior margin strongly raised and subtruncated (Fig. 2A); scutellar shield shorter than elytral suture; internoapical lobe of mesotibia weakly developed and barely defined (Fig. 1H); posterior margin of pygidium narrowly rounded at middle (Fig. 2G)	***M.oblonga* Burmeister**
–	Clypeus densely punctate at apex, with anterior margin weakly raised and rounded (Fig. 2B); scutellar shield longer than elytral suture; internoapical lobe of mesotibia strongly developed and well defined (Fig. 1G); posterior margin of pygidium evenly rounded (Fig. 2H)	***M.olivieri* (Waterhouse)**
23	Inner metatibial spur evenly and strongly curved (Fig. 1K)	***M.chloraspischloraspis* Laporte**
–	Inner metatibial spur straight or barely curved (Fig. 1L)	**24**
24	Smaller specimens (length 7–9 mm); anterior margin of clypeus weakly notched medially (Fig. 2C); scutellar shield slightly constricted basolaterally	***M.xanthosticta* Burmeister**
–	Larger specimens (length 10–11.7 mm); anterior margin of clypeus not notched; scutellar shield more strongly constricted basolaterally	***M.martineziauzerali* Soula**

### ﻿Species treatments

#### ﻿ Macraspis buehrnheimi

Taxon classificationAnimaliaColeopteraScarabaeidae

Bento, Jameson & Seidel sp. nov.

3E9A836F-7D0F-594A-B3F0-259B6D80B1CE

https://zoobank.org/EFC64A56-50AF-4C02-84AD-E6D05ECF3871

Figs 3A–G , 4A–D , 5A–C , 12

##### Type material

**(1 male, 1 female). *Holotype*** male deposited at CZPB, labeled: “BRASIL, Amazonas, Coari, / rio Urucu, LUC – 09, 4°51'56"S, 65°04'56"W, / 25/I–10/II/1995, P. F. / Bührnheim et al col.” (white, printed) // “à luz mista / de mercúrio” (white, printed) // “HOLOTYPE / Macraspis buehrn-/ heimi Bento, Jameson, / Seidel, 2022 / M. Bento, det. 2022. ***Paratype***: same data as holotype (1 ♀, CZPB).

##### Diagnosis.

Male genitalia are required for identification: lateral articular areas of tectum pointed and strongly projected distally (Fig. 3E); paramera in dorsal view slightly constricted medially, with middle portion almost as wide as apical portion (Fig. 3E); apex of paramera in caudal view obtusely triangulate, with a strong median tooth (Fig. 3F, G).

##### Description.

**Holotype male** (Fig. 3A, B, E–G). Length 10.6 mm, width 5.9 mm. Body rounded-oval. ***Coloration*.** Head, elytra, and scutellar shield shiny green, with brownish reflections. Pronotum shiny green, anterolateral areas with brownish reflection, and posterolateral areas with yellow maculae laterally extending to anterior margins. Pygidium and venter shiny green with strong brownish reflections. ***Head*.** Vertex sparsely punctate on disc, laterally punctostriate. Frons with slight V-shaped depression, moderately punctate, punctures moderate and deep. Interocular width 3.7 times wider than transverse eye diameter. Clypeus confluently punctate, with anterior margin subtrapezoidal, slightly raised medially. Mandible with outer teeth strongly raised, outer margin slightly curved near base. ***Pronotum*** shallowly and sparsely punctate on disc, punctures small and shallow; anterolaterally punctostriate, punctures large and deep. ***Scutellar shield*** moderately punctate, longer than elytral suture. ***Elytra*** 2 times longer than mid-width, moderately punctate, punctuations large and shallow. Posthumeral depression well developed laterally. Apical umbone wide and poorly defined. ***Pygidium*** strongly convex, with weak and concentric sculpturing, slightly effaced posteriorly. ***Venter*** glabrous, moderately punctate. Mesometaventral process anteriorly directed between procoxae, ventrally flat, with apex abruptly acute in anteroventral view. Mesepimera partially exposed in dorsal view, strongly convex and transversally ridged. ***Legs*.** Protibia externally tridentate, with proximal tooth well defined and acute. Protarsomere V longer than protarsomeres I–IV combined. Anterior protarsal claw enlarged, unequally bifid and obliquely truncated. Mesotibia with internal margin straight, with inner apex not dilated. Mesotarsomere IV with ventroapical projection well developed, thickened and ventrally swollen. ***Abdomen*** with ventrite 6 broadly and slightly emarginated posteriorly. ***Aedeagus*** (Fig. 3E–G). Tectum abruptly narrowed towards the apical edge, with lateral articular areas pointed and strongly projected distally. Paramera in dorsal view slightly constricted medially, with middle portion almost as wide as apical portion; apex in caudal view triangulated with a strong median tooth, strongly deflected ventrally. ***Endophallus*** (Fig. 4A–D) divided into three portions: one narrow, tube-shaped basal portion; one wide, sac-shaped medial portion; and one hairy, slender apical portion (partially lost in Fig. 4A, B, D). Proximal portion distally hairy; V-shaped sclerite with thin, long arms; and temones large, fused into a single sclerite with a mediolongitudinal carina. Medial portion with a broad ventral raspula and a small dorsomedial raspula bearing moderately dense, thin-walled asperites; a dorsodistal raspula bearing multiple, irregular, and dense rows of thick-walled asperites; and a large, triangular lateral sclerite, with distal edge thick and slightly raised.

**Paratype (1 female) (Fig. 3C, D).** Length 11.2 mm. Width 6.3 mm. The female differs from male by the more robust and more convex body; interocular width 4.2 times wider than transverse eye diameter; clypeus longer, with anterior margin narrower and more raised; pygidium plano-convex; protibia with outer teeth stronger and apically rounded; Mesotarsomere IV with a short ventroapical projection straight and pointed; and abdominal ventrite 6 not emarginated. ***External genitalia*** (Fig. 5A–C). Gonocoxites dark brown, strongly sclerotized and moderately setose apically, setae moderately long. Proximal gonocoxites rugostriate and large, as long as wide, overlapping the distal gonocoxites; inner margin abruptly deflected to apex narrow. Distal gonocoxites with inner margin curved and apex narrowly rounded.

##### Etymology.

This species is named after the Brazilian zoologist Paulo Friederich Bührnheim (1937–2001), who greatly contributed to education and research in Amazonas state, Brazil. In addition, he founded the insect collection at the Universidade Federal do Amazonas (UFAM) and collected the type series of this species.

##### Distribution

**(Fig. 12).** Brazil (2). Amazonas: Coari.

##### Remarks.

*Macraspisbuehrnheimi* sp. nov. has the same color pattern as *M.lateralis* (Olivier, 1789), *M.fernandezi* Neita-Moreno, 2014, and *M.phallocardia* sp. nov. These species are only separated by careful comparison of male genitalia. The male aedeagus of *M.buehrnheimi* is most similar to that of *M.fernandezi* (unknown female) in that both have paramera apically dilated. Other characters that serve to separate *M.buehrnheimi* and *M.fernandezi* are (characters of *M.fernandezi* given in parenthesis): tectum abruptly narrowed towards the apical edge, with lateral articular areas pointed and strongly projected distally (tectum evenly narrowed towards the apical edge, with lateral articular areas truncated and weakly projected distally (Fig. 3H)); paramera in dorsal view slightly constricted medially, with middle portion almost as wide as apical portion (paramera in dorsal view strongly constricted medially, with middle portion narrower than half the apical portion (Fig. 3H, I)); apex of paramera in caudal view triangulated, with a strong median tooth (apex in caudal view oblong-oval, with a weak median tooth (Fig. 3I)).

There are no reliable means to distinguish the female of *M.buehrnheimi* sp. nov. from that of *M.phallocardia* sp. nov. based on external morphology. Analysis of the external genitalia showed conspicuous differences in the proximal and distal portions of the gonocoxites of these species (compare Fig. 5A, B to Fig. 5D, E). However, the scarce number of specimens prevented us from assessing intraspecific variation in these structures, which need further morphological examination within the genus. Females of these species should be reliably identified when collected with associated males.

#### ﻿ Macraspis fernandezi

Taxon classificationAnimaliaColeopteraScarabaeidae

Neita-Moreno, 2014

6209BC03-9542-5304-AE0F-D2B0C1AA67E9

Figs 1O , 3H–J , 4E–G , 12

##### Type material examined.

***Holotype*** male deposited at IAVH, labeled: “COLOMBIA, Meta, PNN / La Macarena San Juan de / Arama 03°20'47"N, 73°53'22"W Caño La Curia / 580 m Bos. Galeria 13.iii.1986 / F. Fernández leg.” (white, printed; duplicated label) // “*Macraspis fernandezi* / Neita-Moreno 2014” (white, printed) // “HOLOTIPO / *Macraspis fernandezi* / Neita-Moreno, 2014” (red, printed) // “Instituto Humboldt / Colombia / IAvH-E-88479” (white, printed with added QR-code).

##### Additional material examined

**(2 males).** Brazil, Roraima, Caracaraí, Parque Nacional da Serra da Mocidade, 15–26.I.2016, F. F. Xavier, R. Boldrini, & P. Barroso (legs.) (♂, INPA); Brazil, Amazonas, Manaus, Fazenda Dimona – PDBFF, 19.VIII.2000, R. Andreazze (leg.) (♂, INPA).

##### Distribution

**(Fig. 12).** Colombia (1). Meta (Neita-Moreno 2014). Brazil (2). Roraima, Amazonas.

##### Remarks.

This species was described based on a single male specimen from Colombia (Neita-Moreno 2014). Herein, two additional specimens of *M.fernandezi* from Roraima and Amazonas states are recorded for the first time from Brazil (new country record). Further comparison to the holotype male showed that the paramera of Brazilian specimens are slightly narrower at middle, but they do not differ in the lateral articular areas of the tectum. The male endophallus (Fig. 4E–H) of the two Brazilian specimens were inflated and showed no conspicuous intraspecific differences. Female specimens of this species have not been described.

#### ﻿ Macraspis lateralis

Taxon classificationAnimaliaColeopteraScarabaeidae

(Olivier, 1789)

F08D8D80-8CF2-5F46-9A36-1D04E7FB1650

**Figure 6.**
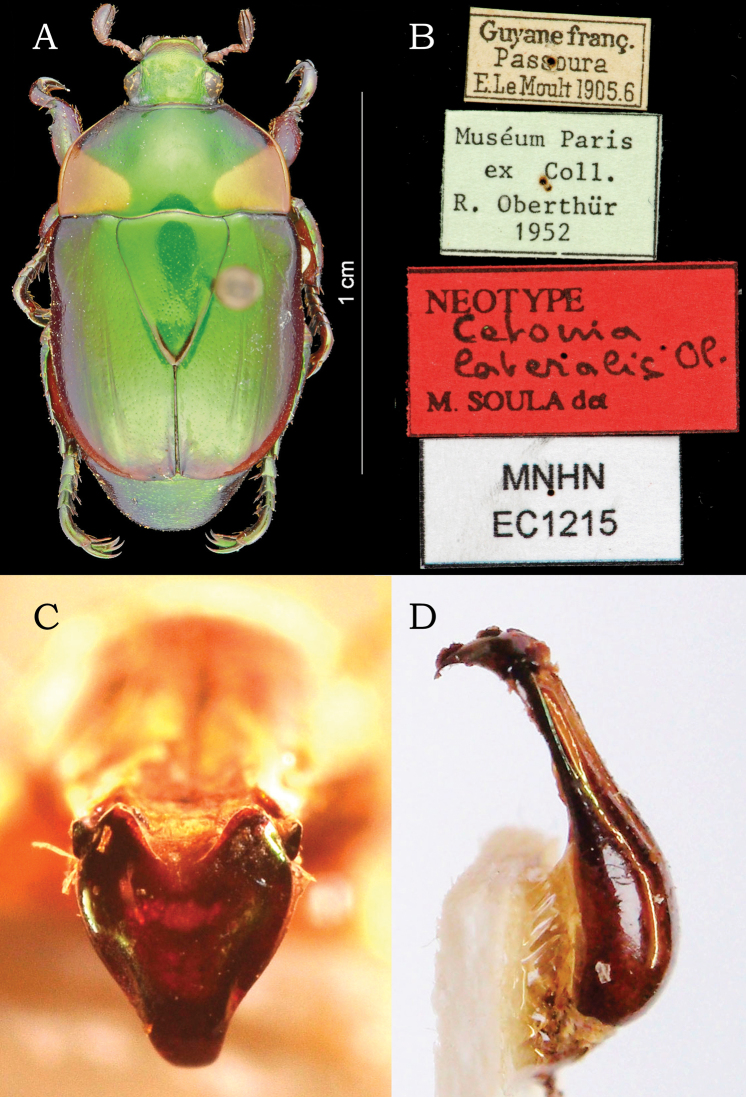
Neotype male of *Cetonialateralis* Olivier, 1789 (=*Macraspislateralis* (Olivier)): **A** dorsal view **B** labels **C** aedeagus in frontal view **D** aedeagus in lateral view. **A, B** from MNHN website http://coldb.mnhn.fr/catalognumber/mnhn/ec/ec1215, accessed on 2022-07-23; **C, D** provided by Jhon César Neita-Moreno.

**Figure 7.**
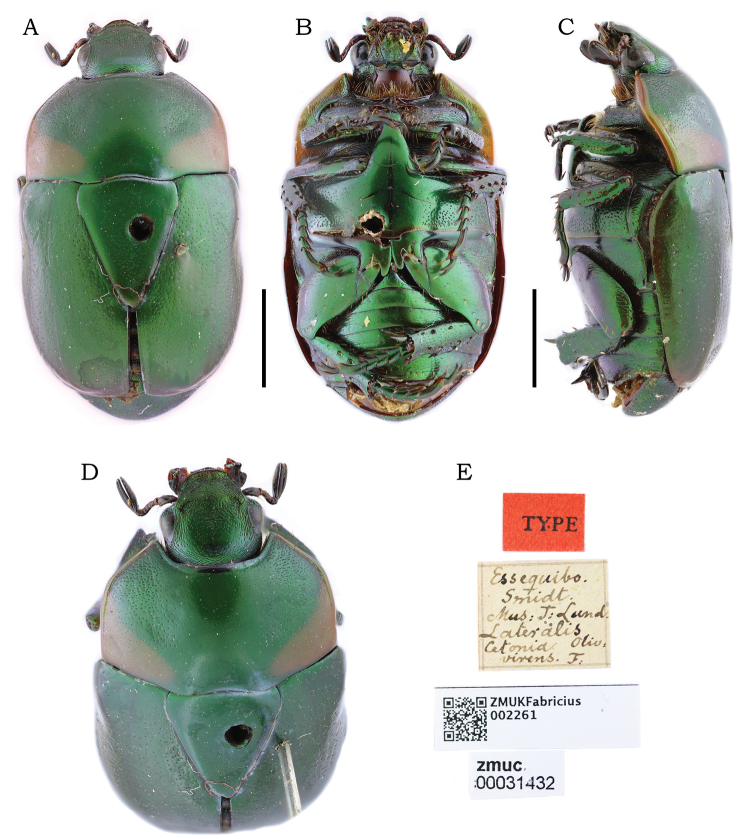
Holotype female of *Cetoniavirens* Fabricius, 1801 (=*Macraspislateralis* (Olivier)): **A** dorsal view **B** ventral view **C** lateral view **D** head and pronotum in dorsal view **E** labels. Scale bars: 2 mm.

Figs 6A–D , 7A–E , 10H–J , 11A–D , 12

Cetonia lateralis Olivier, 1789: 80.
Cetonia virens Fabricius, 1801: 141. Junior subjective synonym by Burmeister (1844: 350).
Macraspis lateralis var. cincticollis Ohaus, 1898: 52. Junior subjective synonym by Machatschke (1972: 71).
Macraspis lateralis var. immaculata Ohaus, 1898: 52. Junior subjective synonym by Machatschke (1972: 71).

##### Type material examined.

***Neotype*** male (Fig. 6) deposited at MNHN, labeled: “Guyane franç. / Passoura / E.LeMoult 1905.6 // Muséum Paris / ex Coll. / R. Oberthür / 1952 // NEOTYPE / Cetonia / lateralis Ol. / M. SOULA det // MNHN / EC1215” (http://coldb.mnhn.fr/catalognumber/mnhn/ec/ec1215). Images of the male aedeagus provided by Jhon César Neita Moreno and Julián Clavijo-Bustos (IAVH). ***Holotype*** female of *Cetonia virens* (Fig. 7) deposited at ZMUK, labeled: “Essequibo. / Smidt. / Mus: J: Lund. / Lateralis / Cetonia Oliv. / virens. F.” (white, handwritten) // “TYPE” (red, printed) // “ZMUKFabricius / 002261” (white, printed with QR code) // “zmuc / 00031432” (white, printed).

##### Non-type material examined

**(2 males, 2 females).** Brazil, Amazonas, Manaus, Reserva Ducke, 21.II.1978, Jorge Arias (leg.) (♀, INPA); idem, but no date and collector (♂, INPA); idem, but campus UA, 07.III.2005, Herbert & Marcos Pantoja (legs.) (♂, INPA); Brazil, Pará, Santarém, I.1922, J. F. Zikán Coll. (♀, CEIOC).

##### Distribution

**(Fig. 12).** Suriname (Hielkema and Hielkema 2019). French Guiana. Guiana. Venezuela (Soula 1998, 2002, 2005). Brazil. Amazonas, Pará (Ohaus 1898; present paper).

##### Remarks.

Fabricius (1801) described *Cetoniavirens* from “America Meridionali” (= South America). This species was synonymized under *M.lateralis* by Burmeister (1844), and the synonymy was maintained by Soula (2003) without any additional statements. One of us (MS) located and examined the female holotype of *C.virens* at ZMUK (Fig. 7A–E). Inspection of the label, which was never been completely transcribed, revealed additional, important information. The word “Essequibo” (Guiana), which was omitted from the original description and all treatments of this species, allowed us to ascertain that the type locality was from Guiana. The female holotype is not associated with male specimens so it cannot be reliably identified. However, because the distribution of *C.virens* lies within the known range of *M.lateralis* in the Guiana Shield, we maintain the synonymy.

#### ﻿ Macraspis maculata

Taxon classificationAnimaliaColeopteraScarabaeidae

Burmeister, 1844

6588A24D-1B26-59B9-ADD4-6C0850DDA833

**Figure 8.**
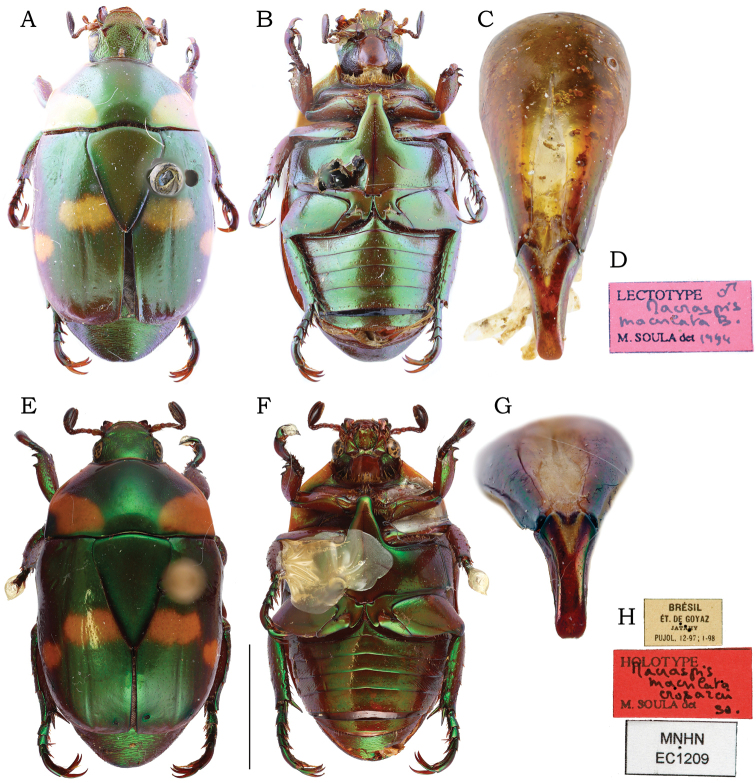
*Macraspismaculata* Burmeister, 1844, lectotype male (**A–D**) **A** dorsal view **B** ventral view **C** aedeagus **D** lectotype label. *M.maculatacrosarai* Soula, 1998, holotype male (**E–H**) **E** dorsal view **F** ventral view **G** aedeagus **H** labels.

**Figure 9.**
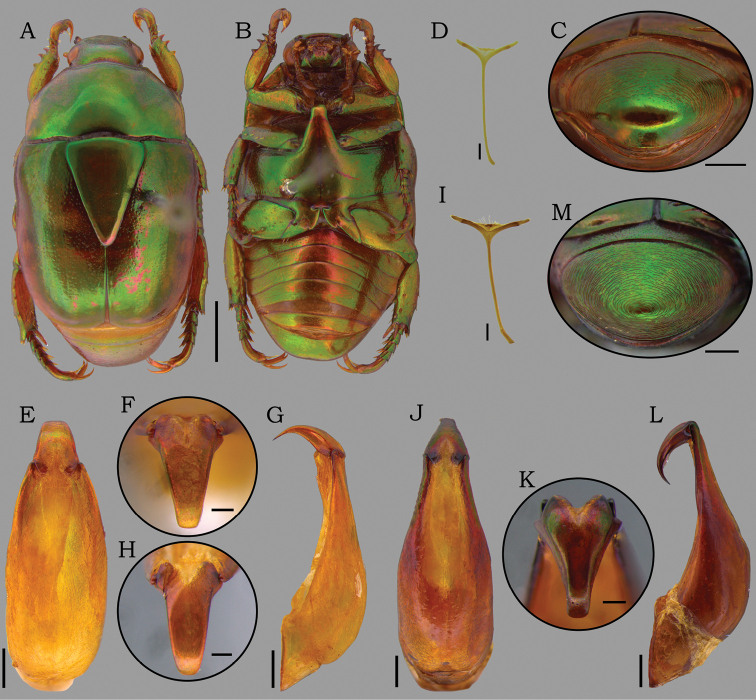
*Macraspisopala* sp. nov. (**A–H**) and *Macraspismaculata* Burmeister (**I–M**): *Macraspisopala* sp. nov., holotype male **A** dorsal view **B** ventral view **C** pygidium in caudal view **D** spiculum gastrale **E** aedeagus, dorsal view **F** aedeagus, caudal view **G** lateral view, paramera (of paratype) and **H** caudal view. *Macraspismaculata* Burmeister, 1844 from Linhares, Espírito Santo state, Brazil, **I** spiculum gastrale **J** aedeagus in dorsal view **K** caudal view and **L** lateral view **M** pygidium in caudal view. Scale bars: 2 mm (**A, B**); 1 mm (**C, M**); 0.5 mm (**E, G, J, L**); 0.2 mm (**F, H, K**).

Figs 2E , 8A–D , 9I–M , 12

Macraspis maculata Burmeister, 1844: 351 (original description); Lucas 1857: 124, pl. 7 fig. 6; Ohaus 1898: 52; 1918: 55; 1934: 154; Blackwelder 1944: 242; Machatschke 1972: 71; Soula 1998: 37; 2003: 39; Krajcik 2007: 80; Brûlé et al. 2014: 182; Ratcliffe et al. 2015: 199; Hielkema and Hielkema 2019: 143.
Macraspis maculata crosarai Soula, 1998: 38 (original description); Soula 2003: 39; Krajcik 2007: 80. Syn. nov.

##### Type material examined

**(2 males, 1 female). *Lectotype*** male of *Macraspismaculata* (designated by Soula, 1998) deposited at MLUH, labeled: “maculata / Dej. / Bras. Coll. (green, handwritten) // LECTOTYPE ♂ / Macraspis / maculata B. / M. Soula 1994”. ***Holotype*** male of *Macraspismaculatacrosarai* Soula deposited at MNHN, labeled: “BRÉSIL / ÉT. DE GOYAZ / JATAHY / PUJOL, 12-97; 1-98” (white, printed) // “HOLOTYPE / Macraspis / maculata / crosarai / So. / M. Soula det.” (red, printed and handwritten). ***Paratype*** female of *M.maculatacrosarai* at MNHN, labeled: “Jathay / État de Goyaz / Ch. Pujol 1895-96” (white, printed) // “Muséum Paris / ex Coll. / R. Oberthür / 1952” (bluish white, printed) // “MACRASPIS / maculata Bu / Soula det.” (white, printed) // “ALLOTYPE / Macraspis / maculata / crosarai / So. / M. Soula det.” (red, printed) // “MNHN / EC10776” (white, printed).

##### Additional material examined

**(18 males, 13 females).** Brazil, Bahia, Prado, 05.III.1971, C. Elias (leg.) (♀, DZUP); Brazil, Espírito Santo, Conceição da Barra, 04.X.1969, C. T. & C. Elias (legs.) (♂, ♀, DZUP); idem, but 26.IX.1968 (2♂, ♀, DZUP); idem, but 19–25.XI.1968 (♂, ♀, DZUP); Brazil, Espírito Santo, Linhares, X.1965 (♂, DZUP); idem, but XI.1965 (♀, DZUP); idem, but no date (♂, DZUP); idem, but XI.1966, A. Maller (leg.) (♂, ♀, DZUP); idem, but Rio Itabapoana, 26.X.1906, J. F. Zikán Coll. (♀, CEIOC); Brazil, Rio de Janeiro, Rio de Janeiro, Corcovado, XII.1961, Seabra & Alvarenga (legs.) (♀, DZUP); Brazil, Minas Gerais, Rio José Pedro, 28.XI.1926, J. F. Zikán Coll. (♀, CEIOC); Brazil, Tris. [= Trisanga (= Irisanga)], N.8 (3♂, NHMW).

##### Distribution

**(Fig. 12).** Brazil. São Paulo (Irisanga = Oriçanga; Burmeister 1844), Rio de Janeiro, Goiás (Soula 1998, 2003), Bahia, Espírito Santo, Minas Gerais (new state records).

##### Remarks.

*Macraspismaculata* has been recorded from the Brazilian Amazon (Ohaus 1918, 1934; Machatschke 1972; Brûlé et al. 2014) but similarities with other species have posed problems with identification. In his treatment for *M.maculata*, Soula (1998) considered the species to be widely distributed, and he justified the creation of related taxa because: “… some populations that are more geographically isolated are already genetically isolated enough to describe subspecies (or even new species ...)” (translated from French). Soula (1998, 2003) restricted *M.maculatamaculata* to Brazilian Atlantic Forest and described *M.maculatacrosarai* from Brazilian Cerrado (distinguished from the nominotypical subspecies by means of coloration). Influenced by Antonio Martínez’s identification labels, Soula (1998) described *M.martinezi* and compared it with *M.maculata* (perhaps constituting one of Soula’s “genetically isolated” populations of *M.maculata*). He included three subspecies, *M.martineziauzerali*, *M.martinezicuroei* Soula, 1998, and *M.martinezicolombica* Soula, 1998, all of which are distributed in the Amazon biome (only the first subspecies is known from the Brazilian Amazon) and similar to *M.maculata* in coloration, form, and genitalic form. The validity of *M.martinezi* and its subspecies-complex requires evaluation in future studies. Examination of the primary types of *M.maculatamaculata* (Fig. 8A–D) and *M.maculatacrosarai* (Fig. 8E–H) revealed no conspicuous differences between these taxa. Based on this examination, we synonymize *M.maculatacrosarai* with the nominotypical *M.maculata*. Now, *M.maculata* no longer includes subspecies. Therefore, although the taxon has been recorded to the Brazilian Amazon and French Guiana by past authors (Ohaus 1918, 1934; Machatschke 1972; Brûlé et al. 2014), *M.maculata* is treated *sensu*Soula (1998, 2003) in this paper and considered to be distributed in the Brazilian Cerrado and Atlantic Forest rather than the Brazilian Amazon region. For this reason, this species was excluded from the key provided here.

#### ﻿ Macraspis oblonga

Taxon classificationAnimaliaColeopteraScarabaeidae

Burmeister, 1844

DE103152-DEDE-5E5B-B585-2C0B156C4700

Figs 1H , 2A, G , 12

Macraspis oblonga Burmeister, 1844: 359 (original description); Ohaus 1898: 51; 1918: 56; 1934: 154; Blackwelder 1944: 242; Machatschke 1972: 73; Soula 1998: 48, 111; 2003: 47; Krajcik 2007: 81; Hielkema and Hielkema 2019: 144.

##### Material examined

**(4 females).** Brazil, Amazonas, Manaus, Fazenda Esteio, 30.VIII.1984, R. C. Klein (leg.) (♀, INPA); Brazil, Amazonas, BR-174 Km 18, 11.VIII.1980, C. Fonseca & E. Bindá (legs.) (♀, INPA); Brazil, Amazonas, Manaus, Reserva Ducke, 14.VIII.1969, A. Faustino (leg.) (♀, INPA); Brazil, Amazonas, Presidente Figueiredo, 21.IX.1992, Aquino (leg.) (♀, INPA).

##### Distribution.

Suriname (Hielkema and Hielkema 2019). French Guiana (Soula 1998). Brazil (Fig. 12). Amazonas (present paper).

##### Remarks.

This species is sympatric with *M.olivieri*, and both have a very similar color pattern. However, *M.oblonga* is easily distinguished from *M.olivieri* by (characters of *M.olivieri* given in parenthesis): clypeal apex rugopunctate, with anterior margin strongly raised and subtruncated (Fig. 2A) (clypeal apex densely punctate, with anterior margin weakly raised and rounded (Fig. 2B)); scutellar shield shorter than elytral suture (longer); internoapical lobe of mesotibia weakly developed and barely defined (Fig. 1H) (strongly developed and well defined); posterior margin of pygidium narrowly rounded at middle (Fig. 2G) (evenly rounded). This is the first record of *M.oblonga* in Brazil (new country record) based on female specimens collected in Amazonas state.

#### ﻿ Macraspis opala

Taxon classificationAnimaliaColeopteraScarabaeidae

Bento, Jameson, Seidel sp. nov.

0861E88A-BE06-553B-BFB6-2F743A10BB46

https://zoobank.org/CB4B582C-5434-46BB-A747-51EDD90D4EB1

Figs 9A–H , 12

##### Type material

**(2 males). *Holotype*** male deposited at DZUP, labeled: “Brasil – Pará / Itaituba / Rio Tapajós” (white, printed) (verse: “RU / 65 ♂. / 2.65.” (handwritten)) // “DZUP/530613” (white, printed) // “HOLOTYPE / Macraspis opala / Bento, Jameson, Seidel, / 2022 / M. Bento, det. 2022”. ***Paratype*** male, labeled: “Brasil. Rondônia – UHE / Samuel – Canteiro de / obras. 07–08/XI/1986 / J. C. Costa e Z. F. Silva, col.” (white, handwritten) (CZPB).

##### Diagnosis.

Apex of pygidium smooth (Fig. 9C); metatarsomere IV with ventroapical projection short and apex acute; apical edge of tectum wider than base of paramera, with lateral articular areas horizontally rotated (Fig. 9E–H); paramera strongly declivous laterally, with sides inconspicuous in caudal view and strongly excavated medially (Fig. 9F–H).

##### Description.

**Holotype male (Fig. 9A–G).** Length 9.7 mm, width 5.6 mm. Body rounded-oval. ***Coloration*.** General color copper with green or red reflections. Head, pronotum, and scutellar shield with strong green reflections. Pronotum with yellow posterolateral maculae effaced. Elytra brownish copper, with two median yellow maculae somewhat effaced. Pygidium and venter with color more diffuse than dorsal surface. ***Head*.** Vertex sparsely punctate at disc, laterally punctostriate. Frons with slight V-shaped depression, densely punctate, punctures large. Interocular width 4.2 times wider than transverse eye diameter. Clypeus confluently punctate, with anterior margin subtrapezoidal, slightly raised medially. ***Pronotum*** shallowly and sparsely punctate at disc, with slight anterolateral depression densely punctate at lateral corner, punctures moderate and deep. ***Scutellar shield*** moderately punctate, longer than elytral suture. ***Elytra*** 1.8 times longer than mid-width, moderately punctate, punctuations moderate and shallow. Posthumeral depression weak. Apical umbone wide and well defined. ***Pygidium*** (Fig. 9C) strongly convex and apically smooth. ***Venter*** glabrous, moderately punctate. Mesometaventral process anteriorly directed between procoxae, ventrally flat, with apex abruptly acute in anteroventral view. Mesepimera partially exposed in dorsal view, slightly convex and transversally ridged. ***Legs*.** Protibia strongly tridentate externally, with proximal tooth well defined and pointed. Protarsomere V longer than protarsomeres I–IV combined. Anterior protarsal claw enlarged, unequally cleft and obliquely truncated. Mesotibia with internal margin straight, with inner apex not dilated. Mesotarsomere IV with ventroapical projection well developed, thickened and ventrally curved. Metatarsomere IV with spine-like ventroapical projection short and pointed. ***Abdomen*** with ventrite 6 broadly and slightly emarginated posteriorly. ***Spiculum gastrale*** (Fig. 9D) Y-shaped with proximal arms short and distal stem slender, about 2.8 times longer than arm length. ***Aedeagus*** (Fig. 9E–G). Tectum broadly curved laterally, not narrowed towards the apical third; apical edge wider than base of paramera, with lateral articular areas horizontally rotated. Paramera strongly declivous laterally, with sides inconspicuous in caudal view and strongly excavated medially to form a laterolongitudinal carina.

**Female.** Unknown.

**Paratype (1 male).** The male paratype differs from holotype by the general coloration darker with stronger green reflections and elytra without yellow maculae; paramera slightly narrower, with lateral margins parallel and apex slightly more rounded (Fig. 9H).

##### Etymology.

The specific epithet is derived from the Latin word ‘*opalus*’ (= precious stone) in reference to opal gemstone, alluding to the metallic, multicolor cuticular surface.

##### Distribution

**(Fig. 12).** Brazil (2). Pará: Itaituba, Rondônia: Candeias do Jamari. The two male specimens composing the type series were collected within the Madeira-Tapajós interfluvium.

##### Remarks.

This species is quite similar to *Macraspismaculata* Burmeister, 1844. However, *M.opala* sp. nov. has a slightly smaller body size and is distinguished from *M.maculata* by (characters of *M.maculata* given in parenthesis): apex of pygidium smooth, without sculpturing (apex of pygidium with strong and concentric sculpturing (Fig. 9M)); metatarsomere IV with ventroapical projection short and pointed (metatarsomere IV with ventroapical projection thickened and truncated); tectum broadly curved laterally, apical edge wider than base of paramera, with lateral articular areas horizontally rotated (tectum laterally narrowed towards the apical third, apical edge as narrow as base of paramera, with lateral articular area vertically positioned (Fig. 9J–L)); paramera strongly declivous laterally, with sides inconspicuous in caudal view and strongly excavated medially to form a laterolongitudinal carina (paramera slightly declivous laterally, with sides conspicuous in caudal view and weakly excavated apically (Fig. 9K, L)).

#### ﻿ Macraspis phallocardia

Taxon classificationAnimaliaColeopteraScarabaeidae

Bento, Jameson, Seidel sp. nov.

1DFAB378-0AE1-562E-85F8-9A42ED723B33

https://zoobank.org/315D05D9-1487-4BEC-8F7D-730DF26DC6FB

**Figure 10.**
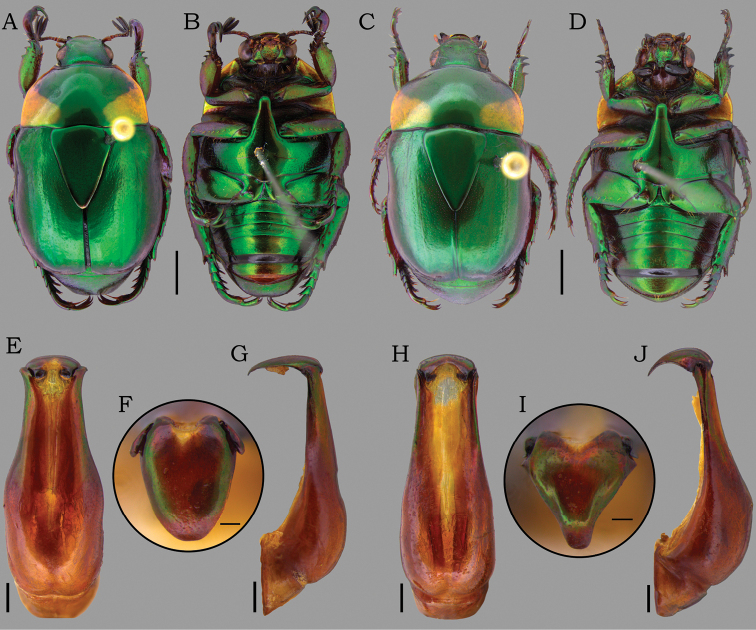
*Macraspisphallocardia* sp. nov. (**A–G**) and *Macraspislateralis* (Olivier, 1789) (**H–J**). *Macraspisphallocardia* sp. nov. **A** holotype male dorsal view **B** holotype male ventral view **C** paratype female dorsal view **D** paratype female ventral view **E–G** aedeagus of holotype in **E** dorsal view **F** caudal view **G** lateral view. *Macraspislateralis* (Olivier, 1789) from Manaus, Amazonas state, Brazil **H** aedeagus in dorsal view **I** caudal view **J** lateral view. Scale bars: 2 mm (**A–D**); 0.5 mm (**E, G, H, J**); 0.2 mm (**F, I**).

**Figure 11.**
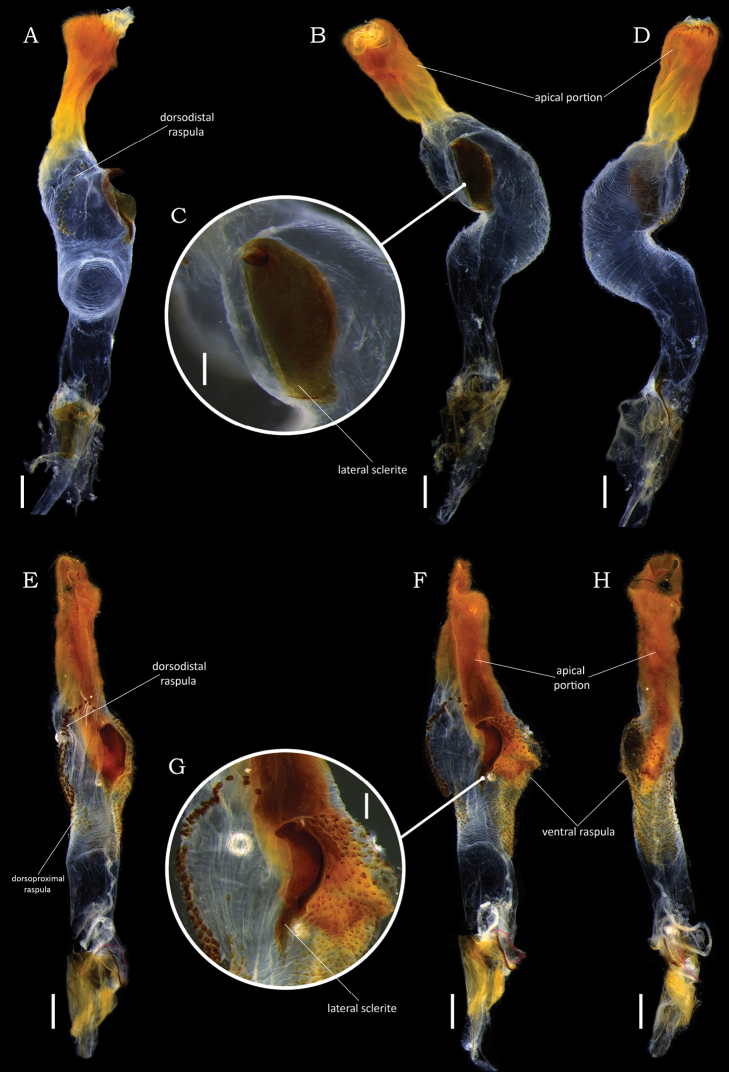
Comparison of male endophalli of *Macraspislateralis* (Olivier, 1789) (**A–D**) and *Macraspisphallocardia* sp. nov. (**E–H**): *Macraspislateralis* (Olivier, 1789) in **A** dorsal view **B** lateral view **D** lateral view and **C** showing detail of lateral sclerite. *Macraspisphallocardia* sp. nov. in **E** dorsal view **F** lateral view **H** ventral view and **G** showing detail of lateral sclerite. Scale bars: 0.5 mm (**A, B, D, E, F, H**); 0.2 mm (**C, G**).

**Figure 12.**
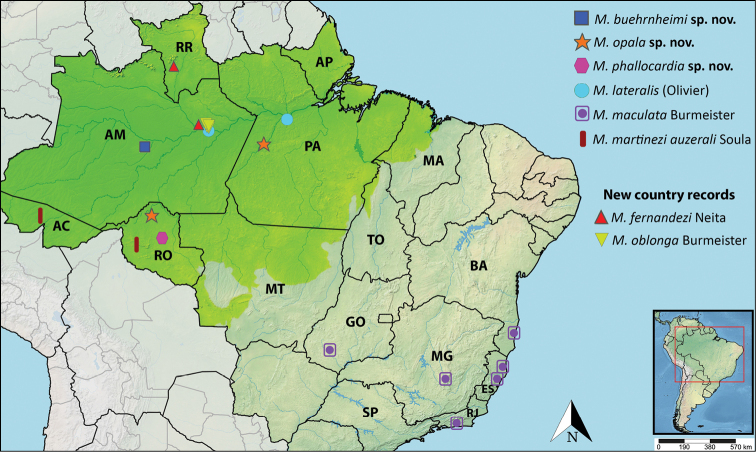
Distribution of Brazilian species of *Macraspis* treated in this publication. The Brazilian Amazon biome is colored lime green. Brazilian states are abbreviated as follows: AC = Acre, AM = Amazonas, AP = Amapá, BA = Bahia, ES = Espírito Santo, GO = Goiás, MA = Maranhão, MG = Minas Gerais, MT = Mato Grosso = PA = Piauí, RO = Rondônia, RJ = Rio de Janiero, RR = Roraima, SP = São Paulo, TO = Tocantins.

Figs 1L , 5D–G , 10A–G , 11E–H , 12

##### Type material

**(4 males, 14 females). *Holotype*** male deposited at **DZUP**, labeled: “Ouro Preto / d’Oeste, RO / 29-X-1987 / C. Elias, leg” (white, printed) // “Projeto Po / -lonoroeste” (white, printed) // “DZUP / 530600” (white, printed) // “HOLOTYPE / M. phallocardia / M. Bento, det. 2019”. ***Paratypes*.** Same data as holotype, but 05.IX.1987 (2 ♀, DZUP), 12.IX.1987 (♀, DZUP), 20.IX.1987 (♂, CEMT, ♀, DZUP), 27.IX.1987 (♀, CEMT, ♂, DZUP), 10.X.1987 (♂, INPA, 2 ♀, DZUP), 18.X.1987 (♀, INPA, 2 ♀, DZUP), 13.XI.1987 (2 ♀, DZUP), 19.VIII.1987 (♀, DZUP), 03.X.1987 (♀, DZUP).

##### Diagnosis.

Male: lateral articular areas of tectum thickened and deflected outward (Fig. 10E); paramera in caudal view rounded-oval, with apex rounded to slightly constricted and sides not declivous (Fig. 10F). Female: Females of this species are diagnosed in association with male specimens.

##### Description.

**Male holotype** (Fig. 10A, B, E–G). Length 11.3 mm, width 6.1 mm. Body rounded-oval. ***Coloration*.** General color shiny green with brownish reflections. Pronotum with posterolateral yellow maculae laterally extending to anterior margins. ***Head*.** Vertex sparsely punctate at disc, laterally punctostriate. Frons with slight V-shaped depression, densely punctate, punctures moderate and deep. Interocular width 4 times wider than transverse eye diameter. Clypeus confluently punctate, with anterior margin subtrapezoidal, slightly raised medially. ***Pronotum*** shallowly and sparsely punctate at disc and moderately punctate anterolaterally, punctures moderate and deep. ***Scutellar shield*** moderately punctate, longer than elytral suture. ***Elytra*** 2.3 times longer than wide, moderately punctate, punctures large. Posthumeral depression weak. Apical umbone wide and poorly defined. ***Pygidium*** strongly convex, with concentric sculpturing and moderately punctate posteriorly. ***Venter*** glabrous, moderately punctate. Mesometaventral process anteriorly directed between procoxae, ventrally flat, with apex abruptly acute in anteroventral view. Mesepimera partially exposed in dorsal view, slightly convex and transversally ridged. ***Legs*.** Protibia externally tridentate, with proximal tooth well defined. Protarsomere V enlarged, longer than protarsomeres I–IV combined. Anterior protarsal claw enlarged, unequally cleft and obliquely truncated. Mesotibia with internal margin straight, with inner apex not dilated. Mesotarsomere IV with ventroapical projection well developed, thickened and ventrally curved. ***Abdomen*** with ventrite 6 broadly and slightly emarginated posteriorly. ***Aedeagus*** (Fig. 10E–G). Tectum moderately narrowed towards the apical edge, with lateral articular areas thickened and deflected outward. Paramera in caudal view rounded-oval, with apex parabolic and sides not declivous. ***Endophallus*** (Fig. 11E–H) divided into three portions: one narrow, tube-shaped basal portion; one wide, sac-shaped medial portion; and one hairy, slender apical portion. Medial portion with a large ventral raspula and a small dorsoproximal raspula bearing dense, thin-walled asperites; a dorsodistal raspula bearing an irregular, dense multiple rows of thick-walled asperites; and a large and cultrate lateral sclerite, with proximal and distal edges thick and slightly raised.

**Paratypes** (3 males, 14 females): male paratypes differ from holotype in length (10.7–11.7 mm), width (5.8–6.7 mm), and form of the apex of paramera (more round to slightly narrowed). Female paratypes (Fig. 10C, D). Length 10.8–11.8 mm, width 6–6.4 mm. The females differ from males by the clypeus longer, anteriorly narrower and more raised; pygidium plano-convex; protibia with outer teeth stronger and apically rounded; protarsomere V simple, with anterior claw unenlarged and equally cleft; inner metatibial spur apically rounded; Mesotarsomere IV with a ventroapical projection short and straight; and abdominal ventrite 6 not emarginated. ***External genitalia*** (Fig. 5D–F). Gonocoxites light brown, slightly sclerotized and moderately setose apically, setae moderately long. Proximal gonocoxites short and semicircular, wider than long, barely overlapping the distal gonocoxites; surface moderately punctate. Distal gonocoxites broadly rounded apically, with inner margin almost straight.

##### Etymology.

The specific epithet is Greek for ‘*phallos*’ (= penis) and ‘*kardia*’ (= heart), refers to the heart-shaped male paramera of this species.

##### Distribution

**(Fig. 12).** Brazil (10). Rondônia: Ouro Preto d’Oeste.

##### Remarks.

This species has the same color pattern as *M.buehrnheimi* sp. nov., *M.fernandezi*, and *M.lateralis*. The male genitalia of *M.phallocardia* sp. nov. is more similar to that of *M.lateralis*, but these species are differentiated by (characters of *M.lateralis* given in parenthesis): tectum with lateral articular areas thickened and deflected outward (tectum with lateral articular areas compressed and straight; Fig. 10H); paramera in caudal view rounded-oval, with apex rounded to slightly constricted and sides not declivous (paramera in caudal view laterobasally projected backward, with sides slightly declivous and narrowly constricted apically; Fig. 10I); medial portion of endophallus with ventral and dorsoproximal raspulae (Fig. 11E–H), a dorsodistal raspula bearing dense and multiple rows of asperites (Fig. 11E), and a lateral sclerite with proximal and distal edges thin and slightly raised (Fig. 11F, G) (medial portion of endophallus without ventral raspulae, Fig. 11D; with a dorsodistal raspula bearing a sparse and simple row of asperites, Fig. 11A; and a lateral sclerite with medial edge raised and distal edge thickened and roundly protruding, Fig. 11B, C). *Macraspisphallocardia* sp. nov. is apparently sympatric with *M.opala* sp. nov., the type series of which was also collected in the Madeira-Tapajós interfluvium.

## Supplementary Material

XML Treatment for Macraspis buehrnheimi

XML Treatment for Macraspis fernandezi

XML Treatment for Macraspis lateralis

XML Treatment for Macraspis maculata

XML Treatment for Macraspis oblonga

XML Treatment for Macraspis opala

XML Treatment for Macraspis phallocardia
